# An Interleukin-6 Receptor Antibody Suppresses Atherosclerosis in Atherogenic Mice

**DOI:** 10.3389/fcvm.2017.00084

**Published:** 2017-12-22

**Authors:** Koji Akita, Kikuo Isoda, Yayoi Sato-Okabayashi, Tomoyasu Kadoguchi, Kenichi Kitamura, Fumie Ohtomo, Kazunori Shimada, Hiroyuki Daida

**Affiliations:** ^1^Department of Cardiovascular Medicine, Juntendo University Graduate School of Medicine, Tokyo, Japan

**Keywords:** interleukin-6, atherosclerosis, inflammation, IκBNS, dyslipidemia

## Abstract

IκBNS is a nuclear IκB protein which negatively regulates nuclear factor-κB activity. We demonstrated that IκBNS deficiency accelerates atherosclerosis in LDL receptor-deficient (LDLr^−/−^) mice *via* increased interleukin (IL)-6 production by macrophages. Previous studies showed that the increase in IL-6 might contribute to the development of atherosclerotic lesions. However, whether an anti-mouse IL-6 receptor antibody (MR16-1) can protect atherosclerotic lesions in atherogenic mice remains to be elucidated. We investigated atherosclerotic lesions in LDLr^−/−^ and IκBNS^−/−^/LDLr^−/−^ mice after 16 weeks consumption of a high-fat diet. All mice received intraperitoneal injections of MR16-1 or phosphate-buffered saline (PBS) (control) once a week during a high-fat diet consumption. Treatment of MR16-1 yielded no adverse systemic effects, and we detected no significant differences in serum cholesterol levels in either group. The atherosclerotic lesions were significantly increased in IκBNS^−/−^/LDLr^−/−^ compared with LDLr^−/−^ mice (*p* < 0.01) under treatment of PBS. However, MR16-1 treatment abolished the significant difference of atherosclerotic lesions between IκBNS^−/−^/LDLr^−/−^ and LDLr^−/−^ mice. Interestingly, MR16-1 also significantly decreased atherosclerotic lesions in LDLr^−/−^ mice compared with PBS treatment (*p* < 0.05). Immunostaining revealed percent phospho-STAT3-positive cell were significantly decreased in the atherosclerotic lesions of MR16-1 treated both IκBNS^−/−^/LDLr^−/−^ and LDLr^−/−^ mice compared with PBS-treated mice, indicating MR16-1 could suppress atherosclerotic lesions *via* the inhibition of IL-6–STAT3 signaling pathway. This study highlights the potential therapeutic benefit of anti-IL-6 therapy in preventing atherogenesis induced by dyslipidemia and/or inflammation.

## Introduction

Nuclear factor-κB (NF-κB) plays essential roles in mediating immune systems. Cytoplasmic IκB family proteins regulate the transcriptional activity of NF-κB, because excessive activation is detrimental to the host (1). After translocation of NF-κB from the cytoplasm to the nucleus, nuclear proteins that are structurally similar to cytoplasmic IκBs take part in the mediating of NF-κB transcriptional activity, as activators or inhibitors, by associating with NF-κB subunits (2). Thus, the regulatory IκB-like nuclear molecules are described as nuclear IκB proteins. Although IκBNS is one of nuclear IκB proteins, the role of IκBNS in the development of atherosclerosis is poorly understood. Our previous study demonstrated that deficiency of IκBNS induces atherogenesis in LDL receptor-deficient (LDLr^−/−^) mice fed a high-fat diet and increases in interleukin (IL)-6 production by macrophages, indicating that IκBNS plays an important role in the suppression of atherosclerotic lesions *in vivo* (3). However, whether or not increased IL-6 production in IκBNS-deficient LDLr^−/−^ mice accelerates atherogenesis remains undetermined.

Several previous reports indicate that IL-6 could accelerate atherosclerosis. Increased plasma levels of IL-6 were associated with cardiovascular mortality over 5-year independent of other risk factor of atherosclerosis (4, 5). Injection of IL-6 itself accelerated atherosclerosis in apolipoprotein E-null mice and C57Bl/6 mice either (4). The genetic polymorphism in the IL-6 signaling pathway concordantly associates with lifetime lower risks of coronary heart disease (6). However, whether inhibition of IL-6 might be effective for the suppression of atherogenesis remains to be elucidated.

We demonstrated here that treatment of an anti-mouse IL-6 receptor antibody (MR16-1) suppressed atherosclerosis lesion in atherogenic mice *via* the inhibition of IL-6–STAT3 signaling pathway.

## Materials and Methods

### Mice

The generation of LDLr^−/−^ mice that lacked IκBNS (IκBNS^−/−^/LDLr^−/−^) used in this study has been described previously (3). Details of IκBNS-deficient mice were described in the previous report (7). We investigated atherosclerotic lesions in LDLr^−/−^ and IκBNS^−/−^/LDLr^−/−^ mice after 16 weeks consumption of a high-fat diet (MF diet containing 0.5% cholesterol, Oriental Yeast Co.). This study was performed according to the protocols approved by the Juntendo University Board for Studies in Experimental Animals.

### Plasma Lipid Measurement

Plasma total cholesterol, triglyceride, high-density lipoprotein (HDL) cholesterol, and low-density lipoprotein (LDL) cholesterol levels were measured by high-performance liquid chromatography at Skylight Biotech Inc. (Akita, Japan).

### Quantification of Atherosclerotic Lesions

After blood collection, the animals were euthanized by pentobarbital injection, and the heart and aorta were flushed with 0.9% NaCl followed by 4% paraformaldehyde. After perfusion procedure, the aorta was harvested and fixed with 10% neutral-buffered formalin for 48 h, embedded in paraffin, and sectioned from just above the aortic valve throughout the aortic sinus (each 6-µm thickness). We used equally spaced 10 cross sections (100-µm interval) to qualify arteriosclerotic lesions in the aortic sinus for each mouse. The samples were stained with Elastica van Gieson, and then photographed using a BX53 microscope (OLYMPUS, Tokyo, Japan). The luminal, arteriosclerotic lesions and medial areas were calculated using NIH Image J 1.42 (National Institutes of Health, public domain software). Quantification of the atherosclerotic lesions was performed by two blinded observers.

The whole aortas were also stained with Sudan IV. The surface atherosclerotic lesions were expressed as the percent of the lesion area extending from the ascending aorta to the iliac bifurcation.

### Immunohistochemistry

Activation of STAT-3 was detected by phospho-Stat3 (Tyr705) (pSTAT3) staining (1:50; Cell Signaling Technology, #9145). Activation both of pSTAT3 was evaluated for percentage of positive nuclei to total nuclei in the arteries.

### Inhibition of IL-6 by Anti-Mouse IL-6 Receptor Antibody (MR16-1) in Mice

All mice received intraperitoneal injections of phosphate-buffered saline (PBS) or MR16-1 (2 mg) once a week during a high-fat diet consumption. MR16-1 was kindly provided from Chugai Pharmaceutical (Japan).

### Statistical Analysis

Results are shown as mean ± SEM. The two groups were compared using Student’s *t*-test. Differences between groups were analyzed using one-way ANOVA test followed by Bonferroni’s *t*-test to determine statistical differences after multiple comparisons. *p* < 0.05 was considered statistically significant.

## Results

### Treatment of MR16-1 Yielded No Adverse in Mice

**Figure 1:**
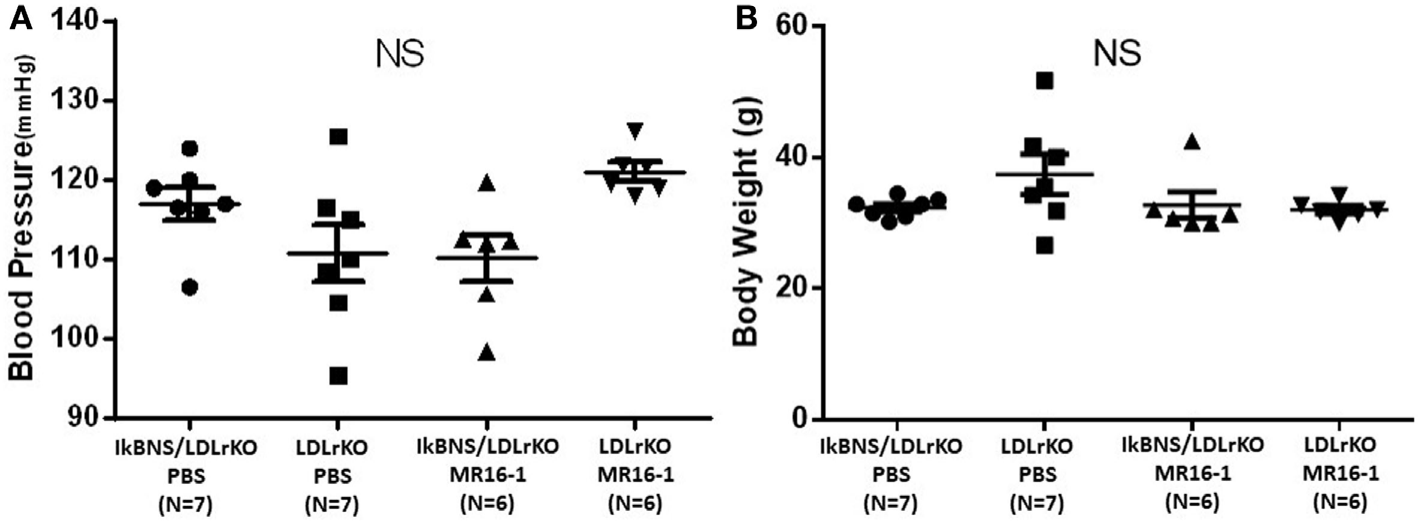
Treatment with MR16-1 did not change blood pressure or body weight in both IκBNS^−/−^/LDLr^−/−^ and LDLr^−/−^ mice. Both mice were received injection of phosphate-buffered saline (PBS) or MR16-1 (2 mg) once a week during a high-fat diet consumption of 16 weeks. Systolic blood pressure **(A)** and body weight **(B)** were similar among four groups. IκBNS/LDLr KO PBS: PBS-treated IκBNS^−/−^/LDLr^−/−^ mice, LDLr KO PBS: PBS-treated LDLr^−/−^ mice. IκBNS/LDLr KO MR16-1: MR16-1-treated IκBNS^−/−^/LDLr^−/−^ mice, LDLr KO MR16-1: MR16-1-treated LDLr^−/−^ mice. Data are expressed as mean ± SEM. NS, not significant.

**Figure 2:**
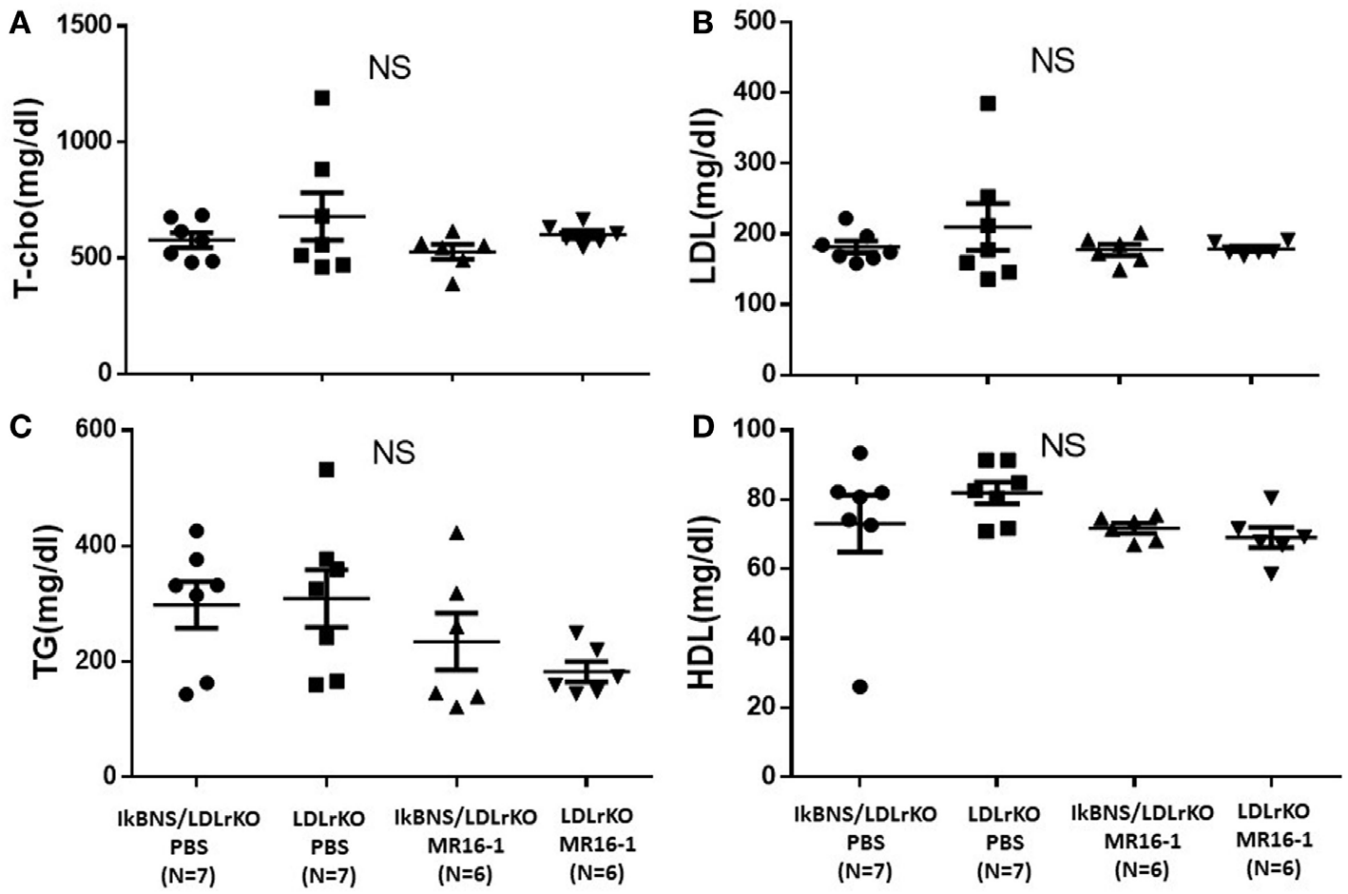
Treatment with MR16-1 did not change cholesterol profiles in both IκBNS^−/−^/LDLr^−/−^ and LDLr^−/−^ mice. The levels of total cholesterol (T-cho) **(A)**, low-density lipoprotein (LDL)-cholesterol **(B)**, triglyceride (TG) **(C)**, and high-density lipoprotein (HDL)-cholesterol **(D)** after treatment of phosphate-buffered saline (PBS) or MR16-1 (2 mg) once a week during a high-fat diet consumption of 16 weeks. IκBNS/LDLr KO PBS: PBS-treated IκBNS^−/−^/LDLr^−/−^ mice, LDLr KO PBS: PBS-treated LDLr^−/−^ mice. IκBNS/LDLr KO MR16-1: MR16-1-treated IκBNS^−/−^/LDLr^−/−^ mice, LDLr KO MR16-1: MR16-1-treated LDLr^−/−^ mice. Data are expressed as mean ± SEM. NS, not significant.

We investigated atherosclerotic lesions in LDLr^−/−^ and IκBNS^−/−^/LDLr^−/−^ mice after 16 weeks consumption of a high-fat diet. Both IκBNS^−/−^/LDLr^−/−^ and LDLr^−/−^ mice received injection of PBS (control) or MR16-1 (2 mg) once a week during a high-fat diet consumption of 16 weeks. MR16-1 treatment yielded no adverse systemic effects. Systolic blood pressure and body weight were similar among four groups (Figures 1A,B). Weight gains of mice after consumption of high-fat diet were similar among the four groups. Moreover, analysis of plasma lipid profiles revealed no statistically significant differences in total cholesterol (Figure 2A), LDL cholesterol (Figure 2B), triglyceride (Figure 2C), and HDL cholesterol (Figure 2D) among these groups.

### MR16-1 Inhibited Atherosclerosis in both LDLr^−/−^ and IκBNS^−/−^/LDLr^−/−^ Mice

**Figure 3:**
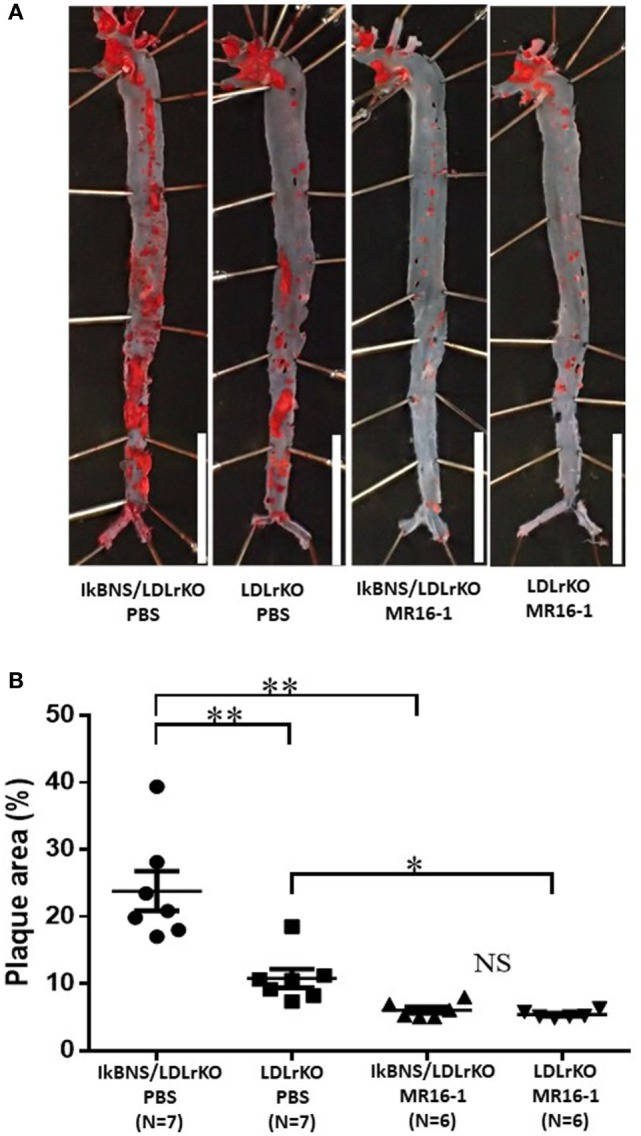
Treatment with MR16-1 decreased in the extent of atherosclerotic lesions in the mice aortas (en face) of both IκBNS^−/−^/LDLr^−/−^ and LDLr^−/−^ mice. **(A)** Sudan IV staining of aortas from four groups after treatment of phosphate-buffered saline (PBS) or MR16-1 (2 mg) once a week during a high-fat diet consumption of 16 weeks (bars = 10 mm). **(B)** Quantitative analysis of the relative surface area of the atherosclerotic lesion in aortas. IκBNS/LDLr KO PBS: PBS-treated IκBNS^−/−^/LDLr^−/−^ mice, LDLr KO PBS: PBS-treated LDLr^−/−^ mice. IκBNS/LDLr KO MR16-1: MR16-1-treated IκBNS^−/−^/LDLr^−/−^ mice, LDLr KO MR16-1: MR16-1-treated LDLr^−/−^ mice. Data are expressed as mean ± SEM. *p < 0.05 and **p < 0.01. NS, not significant.

We investigated the effect of MR16-1 treatment on atherogenesis in both LDLr^−/−^ and IκBNS^−/−^/LDLr^−/−^ mice after 16 weeks consumption of a high-fat diet. As reported previously (3), the extent of atherosclerosis in the mice aortas (en face) was significantly increased in IκBNS^−/−^/LDLr^−/−^ compared with single LDLr^−/−^ mice (*p* < 0.001) under treatment of PBS (Figure 3). However, MR16-1 treatment abolished the significant difference of atherosclerotic lesions between IκBNS^−/−^/LDLr^−/−^ and LDLr^−/−^ mice (Figure 3). Interestingly, MR16-1 treatment also significantly decreased atherosclerotic lesions in LDLr^−/−^ mice compared with PBS treatment (Figure 3).

**Figure 4:**
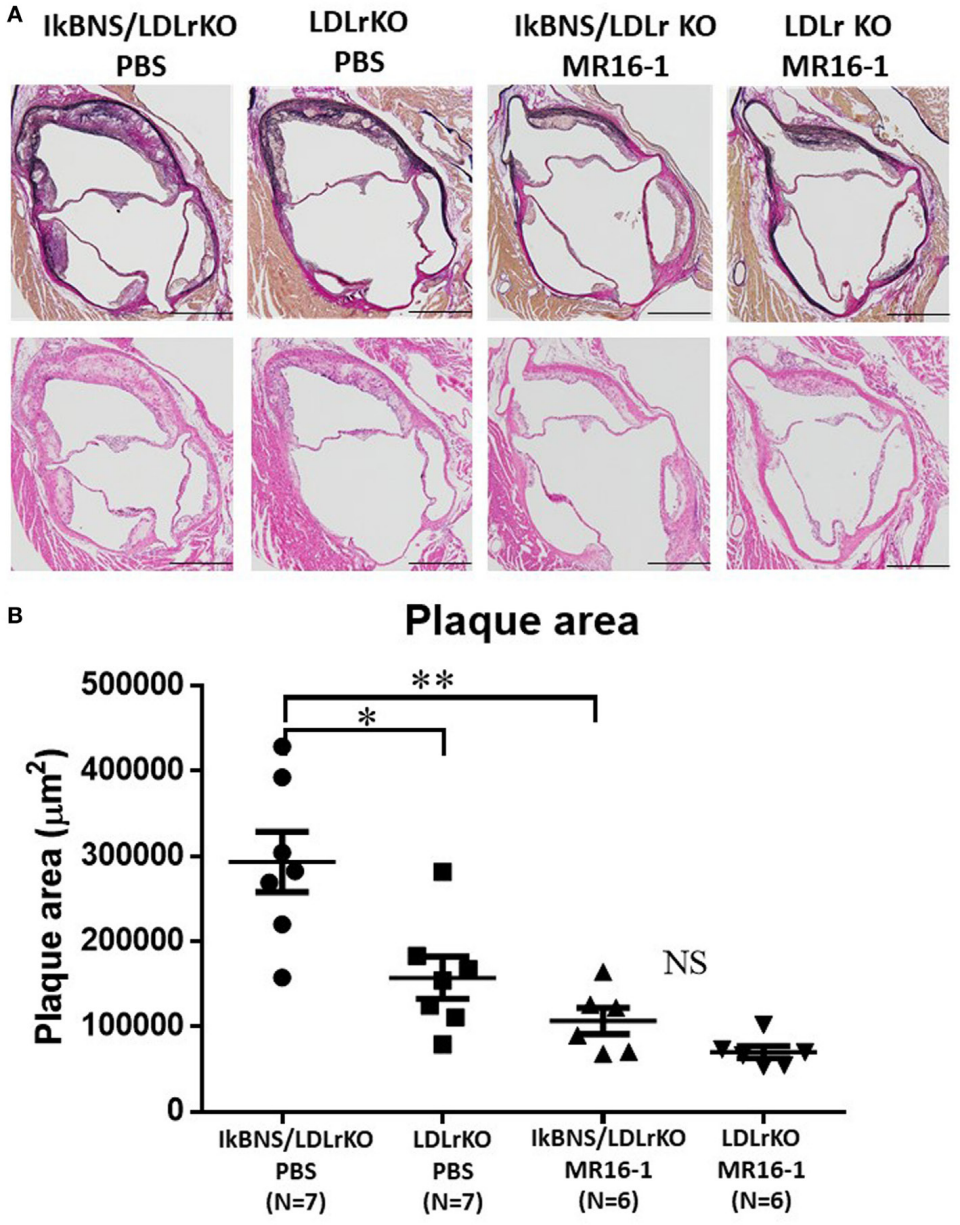
Treatment with MR16-1 decreased in aortic root atherosclerotic lesions of both IκBNS^−/−^/LDLr^−/−^ and LDLr^−/−^ mice. **(A)** Representative photomicrographs of sections of aortic sinus plaque from four groups after treatment of phosphate-buffered saline (PBS) or MR16-1 (2 mg) once a week during a high-fat diet consumption of 16 weeks. Adjacent sections were processed for Elastica van Gieson staining (upper panels) and H&E staining (lower panels) (bars = 500 µm). **(B)** Quantitative comparison of the atherosclerotic lesion in the aortic sinus. IκBNS/LDLr KO PBS: PBS-treated IκBNS^−/−^/LDLr^−/−^ mice, LDLr KO PBS: PBS-treated LDLr^−/−^ mice. IκBNS/LDLr KO MR16-1: MR16-1-treated IκBNS^−/−^/LDLr^−/−^ mice, LDLr KO MR16-1: MR16-1-treated LDLr^−/−^ mice. Data are expressed as mean ± SEM. *p < 0.05 and **p < 0.01. NS, not significant.

Aortic root atherosclerotic lesions of IκBNS^−/−^/LDLr^−/−^ mice were also significantly larger than those in LDLr^−/−^ mice (*p* < 0.05) under treatment of PBS (Figure 4). MR16-1 treatment significantly reduced atherosclerotic lesions in both LDLr^−/−^ and IκBNS^−/−^/LDLr^−/−^ mice compared with PBS treatment (Figure 4).

These results show that an anti-mouse IL-6 receptor antibody significantly suppresses IκBNS in both LDLr^−/−^ and IκBNS^−/−^/LDLr^−/−^ mice without major confounding effects on LDL-cholesterol or HDL-cholesterol, suggesting that inhibition of IL-6 might be significantly effective to prevent atherogenesis.

### MR16-1 Suppressed STAT3 Activation in the Atherosclerotic Lesions of both LDLr^−/−^ and IκBNS^−/−^/LDLr^−/−^ Mice

**Figure 5:**
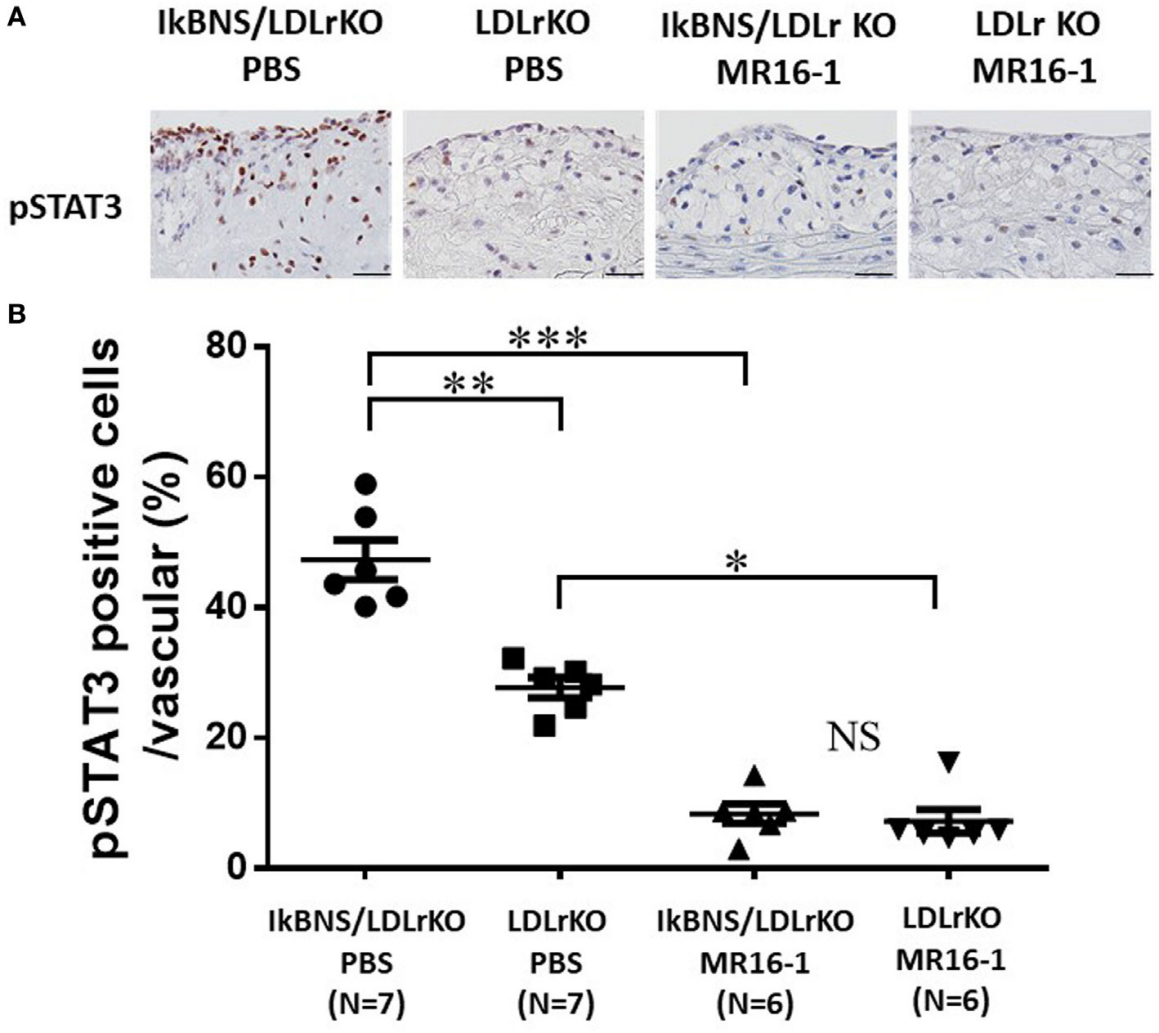
MR16-1 treatment significantly inhibited STAT3 activation in the atherosclerotic lesions of both IκBNS^−/−^/LDLr^−/−^ and LDLr^−/−^ mice. **(A)** Representative photomicrographs of sections of aortic sinus plaque from four groups after treatment of phosphate-buffered saline (PBS) or MR16-1 (2 mg) once a week during a high-fat diet consumption of 16 weeks. Sections were processed for pSTAT3 staining (bars = 25 µm). **(B)** Quantitative analysis of pSTAT3-positive cell in vascular wall in sections from four groups. IκBNS/LDLr KO PBS: PBS-treated IκBNS^−/−^/LDLr^−/−^ mice, LDLr KO PBS: PBS-treated LDLr^−/−^ mice. IκBNS/LDLr KO MR16-1: MR16-1-treated IκBNS^−/−^/LDLr^−/−^ mice, LDLr KO MR16-1: MR16-1-treated LDLr^−/−^ mice. Data are expressed as mean ± SEM. *p < 0.05, **p < 0.01, and ***p < 0.001. NS, not significant.

To examine STAT activation in the atherosclerotic lesions of both MR16-1 and PBS groups, we performed pSTAT3 staining in the atherosclerotic lesions of four groups after 16 weeks consumption of a high-fat diet. We detected much more pSTAT3-positive cells in the atherosclerotic lesions of PBS-treated IκBNS^−/−^/LDLr^−/−^ mice compared with PBS-treated IκBNS^−/−^/LDLr^−/−^ mice (Figure 5A). MR16-1 treatment significantly decreased pSTAT3-positive cells in the atherosclerotic lesions of both LDLr^−/−^ and IκBNS^−/−^/LDLr^−/−^ mice compared with PBS treatment (Figure 5A).

To quantify STAT3 activity, we calculated the percentage of pSTAT3-positive nuclei to total nuclei in the arteries. The percentage of pSTAT3-positive nuclei in the aorta of MR16-1-treated IκBNS^−/−^/LDLr^−/−^ mice was significantly lower than that in PBS treated group (47.3 ± 3.1 versus 8.4 ± 1.5%; *p* < 0.01; Figure 5B). Interestingly, MR16-1 treatment also decreased percentage of pSTAT3-positive nuclei in LDLr^−/−^ mice compared with PBS treatment (27.7 ± 1.5 versus 7.2 ± 1.8%; *p* < 0.01; Figure 5B).

## Discussion

Persistent IL-6 production has been shown to play a critical role in chronic inflammatory disease (8). IL-6 signaling has also been linked to plaque initiation and destabilization (9) and to adverse outcomes in the setting of acute ischemia (10). IL-6 stimulates growth of promoters of macrophage and vascular smooth muscle cells, which are major components of plaque (11–13), and IL-6 expression is also detected in human atherosclerotic lesions (14).These findings indicate that IL-6 participates in the development of atherosclerosis. Thus, targeting IL-6 is a rational approach for atheroprotection. To block the effect of IL-6 on the development of atherosclerosis, we used anti-mouse IL-6 receptor antibody (MR16-1). MR16-1 has proved to be effective in experimental model of arthritis (15) and humanized anti-IL-6 receptor antibody (tocilizumab) has been approved by the Food and Drug Administration for the treatment in the patients with rheumatoid arthritis.

Our previous study demonstrates that IL-6 expression and STAT3 activation were increased in the foam cell rich-atherosclerotic lesions of IκBNS^−/−^/LDLr^−/−^ mice *in vivo* and IκBNS^−/−^/LDLr^−/−^ macrophages produced much higher level of IL-6 than LDLr^−/−^ macrophages *in vitro*. These results indicate deficiency of IκBNS increases in the production of IL-6 in macrophage and an increase of IL-6 contributes to the susceptibility to atherogenesis in IκBNS^−/−^/LDLr^−/−^ mice (3). Consequently, because we believe that the mice were suitable for evaluating the effect of IL-6-blocking therapy in atherogenesis, we used IκBNS^−/−^/LDLr^−/−^ mice in this study. As expected, MR16-1 treatment abolished the significant difference of atherosclerotic lesions between IκBNS^−/−^/LDLr^−/−^ and LDLr^−/−^ mice. Interestingly, MR16-1 treatment also significantly decreased atherosclerotic lesions in LDLr^−/−^ mice compared with PBS treatment.

Interleukin-6 is an upstream inflammatory cytokine key player, propagating the downstream inflammatory response in atherosclerosis (16). Previous study demonstrated that inflammatory gene, such as IL-6 (17), which is known as an inducer of STAT3 was highly expressed in atherosclerotic plaques. Moreover, activation of STAT3 has been detected in the plaque (18), and its activation is involved in the progression of atherosclerotic lesions (19). In this study, we analyzed pSTAT3-positive nuclei to evaluate the inhibitory effect of MR16-1 in IL-6 signaling pathway. MR16-1 inhibited STAT3 activation and development of atherosclerotic lesions in both IκBNS^−/−^/LDLr^−/−^ and LDLr^−/−^ mice, suggesting IL-6 is a main contributor to atherogenesis, and inhibition of IL-6 might be a new method to prevent the development of atherosclerosis.

Our data provide solid evidence for a causal role of the IL-6 pathway in the development of atherosclerosis, raising expectations for ongoing trials testing anti-inflammatory strategies for cardiovascular diseases (20).

Although we could not find any adverse systemic effects including signs of infection in MR16-1-treated mice, further study must be needed for checking any adverse effects of MR16-1 in the immune systems.

In our experiments, levels of IL-6 in plasma and liver were not different between IκBNS^−/−^/LDLr^−/−^ and LDLr^−/−^ mice after consumption of high-fat diet. On the other hand, Kuwata et al. (7) demonstrated intraperitoneally injection of LPS induced significantly high-serum levels of IL-6 in IκBNS^−/−^ mice compared to wild-type (IκBNS^+/+^) mice. These findings suggest that feeding a high-fat diet could induce local increase of IL-6 produced by IκBNS^−/−^ macrophages around the vessels, but not serum levels of IL-6. In our previous study, IκBNS expression was observed in all cell types resident to the plaque microenvironment (i.e., smooth muscle cells, macrophages, and endothelial cells) in LDLr^−/−^ mice, but not in IκBNS^−/−^/LDLr^−/−^ mice. Because main IκBNS expression cells were macrophages, we previously examined the effects of the IκBNS deficiency in macrophages. We also investigated the effect of IκBNS deficiency in smooth muscle cells using small interference RNA, because they play important roles in atherogenesis. However, IκBNS deficiency in aortic smooth muscle cell did not affect in IL-6 production *in vitro*. Further experiments might be needed to clarify which cells are main players in atherogenesis induced by IκBNS deficiency.

In conclusion, this study highlights the potential therapeutic benefit of anti-IL-6 therapy in preventing atherogenesis in the mice with dyslipidemia and/or inflammation.

## Ethics Statement

The studies were performed according to the protocols approved by the Juntendo University Board for Studies in Experimental Animals.

## Author Contributions

KA, KI, YO-S, TK, and KK performed the research. KI, KS, and HD designed the research study. KA, YO-S, TK, KK, and FO contributed essential reagents or tools. KA, KI, YO-S, TK, KK, FO, KS, and HD analyzed the data. KA, KI, YO-S, TK, KK, and FO, KS, and HD wrote the paper.

## Conflict of Interest Statement

The authors declare that the research was conducted in the absence of any commercial or financial relationships that could be construed as a potential conflict of interest.
